# Challenges and Barriers for Accessing Online Education Amongst School Children in an Urban Slum Area of Pune, India

**DOI:** 10.7759/cureus.29419

**Published:** 2022-09-21

**Authors:** Asmita Rannaware, Uzma Shaikh, Abhay Gaidhane, Sonali G Choudhari, Sarju Zilate

**Affiliations:** 1 School of Epidemiology & Public Health, Datta Meghe Institute of Medical Sciences, Wardha, IND; 2 Community Medicine, Byramjee Jeejeebhoy (BJ) Government Medical College, Pune, IND; 3 Community Medicine, School of Epidemiology & Public Health, Jawaharlal Nehru Medical College, Datta Meghe Institute of Medical Sciences, Wardha, IND; 4 Pharmacology, Jawaharlal Nehru Medical College, Datta Meghe Institute of Medical Sciences, Wardha, IND

**Keywords:** continuing education in emergencies, urban slum area, education barriers, school children, online education

## Abstract

Background

COVID-19 has restricted the education of students on a global scale. With the nationwide stay-at-home directives, schools, colleges and universities have been shut down. Online education is a measure for continuing the learning of the students in times of pandemic. However, the school-going children of urban slum areas face challenges in attending online classes. Through this study, we have tried to highlight the problems and challenges faced by the students and their parents from an urban slum area of Mangalwarpeth, Pune for attending online education in times of COVID. The urban slum area mainly consists of people from low socioeconomic backgrounds lacking the necessary resources and supportive environment for an online mode of education. We have observed and recorded the response of the participants in the context of online education in times of COVID and challenges faced by the lower socio-economic strata due to reasons like non-availability of resources, poor internet connectivity, poor understanding, and distractions while classes leading to low attendance.

Methods

A cross-sectional qualitative study was conducted in an urban slum area of Mangalwarpeth, Pune. Data was collected over four months after receiving consent from the parents of the children from the metropolitan slum area of Mangalwarpeth. A structured questionnaire was used. Data was coded on an excel sheet and was transferred to SPSS software version 21 (IBM Corp., Armonk, NY) and was represented in frequency and percentage.

Result

After the data collection and analysis, we found that (according to the modified Kuppuswamy scale for socioeconomic status) around 53% of the study population were from the lower middle class followed by the upper lower class (27.16%), upper middle class (9.87%), lower class (8.64%) and upper class (1.23%). Sixteen percent of participants do not have smartphones available, and 95.5% do not have a laptop required for online classes. A total of 19.5% of the students do not have access to internet services. Eighty-four percent of parents agreed on increased expenses of the internet.

Conclusion

Students from urban slum areas belong to lower socioeconomic classes and face problems while attending online classes like the nonavailability of resources and lack of a supportive environment. There are increased expenses of the internet as a result of the online mode of education, with increased distractions from the surroundings, concentration problems, and less understanding of the subjects. The students cannot interact with their teachers and friends and as a result, their social interaction is reduced. A supportive environment and proper resources are essential for the learning of students to continue education in times of emergencies like pandemics.

## Introduction

The outbreak of COVID-19 has impacted the education of many students and youths across the planet. In India, 320 million students have been affected by various restrictions and nationwide lockdowns due to COVID-19 [1]. As per the United Nations Educational, Scientific and Cultural Organization (UNESCO) report, 140 million primary school students and 130 million secondary school students are affected, which are the two most affected levels in India. The pandemic has resulted in inequalities in the education system due to a lack of a supportive learning environment and resources among school-going children [2].

Students from an urban slum area have limited access to smartphones and online internet services. Even if the resources are available, the parents of the school-going children (owning the gadgets like mobile phones, and laptops) are not present (due to work/office) during the online lectures. Thus, the lack of resources contributes to low attendance in online classes. The study focuses on the challenges and barriers to accessing online education for school children of (class 1 to 7) in an urban slum area of Mangalwarpeth, Pune. It is unclear whether the households can look forward to financial returns to education to offset some of the opportunity and financial costs.

Most families from the urban slum are from lower socioeconomic backgrounds and lack sufficient resources to deal with the given situation of the pandemic. Many students lack the motivation to be a part of online classes. Constant monitoring of the children by their parents is not possible at all because of the household chores and hardships for sustenance faced by them during COVID-19.

Many parents with lower socioeconomic status and education cannot motivate their children to join online classes and help in academic learning [3]. Though private schools provide enough teaching-learning material, there are still gaps in the transfer of learning in online classes due to distractions and a lack of monitoring.

There has been a decrease in academic learning activities, and children are more inclined toward digital media [4]. Many children spend their time playing video games and watching TV. This has brought concern to the overall physical and cognitive development of the children and is also leading to a sedentary lifestyle for children [5].

Owing to the online mode of education there is reduced interaction of the children with teachers and their friends as they cannot see, meet, or play with them [6]. This has also reduced the extracurricular activities like dancing, singing, and participating in competitions. This has affected the peer interaction and social and cognitive development of the children.

Due to the school closure and online education, there is disruption of the routine of the meals - breakfast, lunch, and dinner which earlier were satisfactory because of the lunch hours in schools [7]. Many schools provided midday meal schemes to the children, which satisfied the children’s demand for essential caloric and protein requirements. The pandemic has also led to the question of fulfilling the nutritional requirements of the children [8].

The situation of education in COVID times affected students from the urban slum area due to limited resources and expertise. There are very few studies on the problems of students and parents from urban slum areas during pandemic times in India. So, we conducted a survey of students and parents to study the possible influence of the teaching method of online classes in terms of sociodemographic factors in urban slum areas of Pune.

## Materials and methods

Study design

A cross-sectional study was undertaken in urban slum areas of Mangalwarpeth, Pune city of Maharashtra state to study the challenges and barriers of online education for school children. The study was conducted after receiving the approval from Institutional Ethical Committee, BJGMC, and SGH, Pune. The reference number of approval by the Institutional Ethics Committee is BJGMC/IEC/Pharmac/ND-Dept 1220148-148.

Study population

For the present study, all the school-going children from classes 1 to 7 and their parents are the “target population” to which findings are expected to be applicable. People who were not willing to participate were excluded.

Source of data

The data for the present study was obtained from primary sources through interviews with parents and children. The interview questionnaire was prepared and pilot tested after obtaining due consent (filling of assent form wherever applied) from the parents for interviewing their child. The questionnaire comprises sections enquiring about the availability of resources, increased expenses of the internet, availability of time with parents, and sociodemographic conditions of the family. Modified Kuppuswamy scale was used for determining the socioeconomic status of the family.

Sample size calculation and sampling

The sample size is calculated by taking the prevalence of students not able to access online education as 30% [1] at alpha 5% and a confidence interval of 95%, the estimated sample size for the study is 81. The sampling method used was simple random sampling. There are only three government schools in the area of an urban health center in Mangalwarpeth. The sample population was classified into three homogenous groups, by dividing the total population (615) of students into smaller sub-groups and proportional distribution from each class 1 to 7, is taken to constitute the sample size of 81 from all the three schools.

Data analysis

All responses were tabulated using Microsoft Excel 2007 software (Microsoft® Corp., Redmond, WA). The data was then coded and transferred to SPSS software version 21 (IBM Corp., Armonk, NY). Data were analyzed by using statistical tools like Frequency and Percentages. Tabulation of data was done by explaining the frequency and percentage of the characteristic variable.

## Results

The characteristics of the study participants are shown in Table 1. Total participants were 81 amongst which 35 were male and 46 were female. The age group of 5 to 10 years included 56 participants and the age group of 11 to 15 years included 25 participants. According to the modified Kuppuswamy scale around 53.8% of the study participants of our study were from the lower middle class. Only 1.23% of the participants were from the upper class (Table 2).

**Table 1:** Characteristics of the study participants

Characteristic		Frequency (N=81)	Percentage
Age	5 to 10 years	56	69%
	11 to 15 years	25	31%
Gender	Male	35	43.20%
	Female	46	56.80%

**Table 2:** Socioeconomic status of the study participants according to modified Kuppuswamy Socioeconomic Status (SES) scale.

Socioeconomic status	Percentage
Upper class	1.23%
Upper middle	9.87%
Lower middle	53.8%
Upper lower	27.16%
Lower	8.64%

A total of 57% of the study participants were from the lower middle socioeconomic class and 27.16% of the participants were from the upper lower socioeconomic class.

Access to uninterrupted high-quality data - affordability issue, access to assignments

The study states that 19.5% of the students do not have access to internet services, 95% do not have laptops, and 16% do not have smartphones for attending online classes (Table 3).

**Table 3:** Non-availability of resources

Resources	Nonavailability in percentage
Smartphone	16%
Laptop	95%
Internet services	19.5%

As shown in Table 4, 53.8% of children agreed on physical discomfort due to online class (burning of eyes, backache, headache). Amongst the participants, 51.9% of students did not attend classes regularly. Increased screen time is experienced by 77% of children. About 69.1% of students agreed with decreased physical inactivity. Around 79% of students experienced increased distraction in their surroundings during classes. Seventy-nine percent of children agreed on increased homework than regular school homework (Table 4).

**Table 4:** Effects of online classes on children

Variable	Frequency (n=81)	Percentage (n=100%)
Decreased attendance	39	51.9%
Decreased physical activity	54	69.1%
Change in dietary pattern	64	79%
Physical discomfort	43	53.08%
Decreased social interaction	80	98.76%
Increased screen time	63	77.8%
Decreased extracurricular activities	54	69.1%
Distraction during classes	64	79%
Increased homework	64	79%

There was an increase in internet expenses which was experienced by 68% of study population. Around 76.5% of mothers agreed on an increased workload. Engagement of parents in the online classes, increased stress and burden experienced by 88.9%, 77.8%, and 88.9% of parents, respectively (Table 5). A total of 97.9% of parents and 81% of children do not prefer online classes.

**Table 5:** Effects of online classes on parents

Variable	Frequency	Percentage
Increased internet expenses	68	84%
Increased workload on mothers	62	76.5%
Engagement of one parent	72	88.9%
Increased stress	63	77.8%
Increased burden on parents	72	88.9%

## Discussion

Many studies have been conducted to show the effects of online classes on children and their parents during the times of COVID-19 pandemic. These studies show the effect of the pandemic on lives of the school-going children.

Our study concluded that 16% of participants do not have smartphones available, 95.5% do not have a laptop required for online classes, and 19.5% of the students do not have access to internet services. Eighty-four percent of parents agreed on increased expenses of the internet.

The study named “The Impact of COVID-19 on Education: Insights From Education at a Glance 2020” states that the crisis has resulted in many inadequacies and inequities in our education system from access to the broadbands and computers, smartphones needed for online education, the supportive environment needed to focus on learning. Focusing on children's education in the urban slums is needed for their social and cognitive development [9]. Our study highlights the scarcity or availability of the resources needed for the attendance of online classes in urban-slum areas’ school-going children.

The study “Access to and Exclusion From Primary Education in Slums of Dhaka, Bangladesh” [10] assessed the socioeconomic status, health, and parenteral education characteristics of children for their study. In our study, we also undertook the same characteristics for determining the descriptive socioeconomic characteristics of the parents from the study population. We considered the education of the head of the family, occupation of the head of the family, and total per capita family income for determining the socioeconomic status using a modified Kuppuswamy scale.

A research article by Jena states the impact of COVID-19 on education [11]. The author has stated the positive and negative impacts of COVID-19 on education in which there are variables like increased responsibilities over parents, loss of nutrition due to school closure, and access to the digital world. Our study has taken into consideration the effects like increased responsibilities over parents, and increased expenses over the internet.

Verma and Trivedi in a study concluded that around 80% of the students in their survey were interested in online education [12]. Around 38% of the teachers were interested in online education and 63% of parents were interested in online education. The study population chosen was from the Central Board of Secondary Education (CBSE) students, engineering area which belonged to higher socioeconomic classes. In this study, we observed that 97% of the parents from the urban slum area and 81% of the students did not prefer online education when compared to onsite education.

The study “Policy Brief: Education During Covid-19 and Beyond” states that most of the counties in Europe prefer online education compared to Asian countries. The loss of school meals and other health and nutrition services in the first month of the pandemic affected 370 million children in 195 countries [13]. The study discussed that the closure of the institutions has increased domestic violence with women sharing additional time spent on childcare and households. In our study, we have not considered the point of domestic violence as we were focused on learning through online mode.

Kapasia et al. conducted a study in which 232 students mostly graduate and postgraduate from the University of West Bengal were interviewed online via Google forms [14]. They collected the data regarding variables related to characteristics of study participants, knowledge and attitudes regarding COVID-19, information about online classes, learning status, and opinion regarding online education in form of frequency and percentage. According to this analysis, only 26% of the students attended online classes (N=189). Most students (85%) use android mobile for classes and 14.2% have laptops for the classes. The data on the impact of COVID-19 and educational attendance states that 76% of the students agreed that low income would affect their education in COVID times and 75% agreed that the pandemic may cause educational discontinuation. Volery and Lord in their study collected data from anonymous 47 students enrolled in Global Business 650 during the first semester [15]. The student characteristics in our study included demographic variables, internet at home, and recording of socioeconomic factors.

Zhao et al. [16] conducted an online survey separately on parents, students, and teachers. The results found that 76% of students accepted homeschooling, 69% of parents reported increased screen time, 82% has decreased outdoor activity, 74% of the students thought that they could not interact with their teachers, 63% of students and 57% of parents did not agree. Other variables like distractions and decreased outdoor physical activity were also considered. In our study, we used the same variables, however, the responses to the questions were different.

Around 95% of parents and 98% of students disagreed with the continuation of online education. We have also added the sociodemographic variables so that we calculate the frequency of online attendees in the lower socioeconomic group. The size of our sample and the area of data collection was small. The results thus are different. However, the decreased physical activity, distractions during online classes, and interaction with the teachers and students showed nearly the same percentages.

The National Sample Survey (NSS) 75th round on “Key Indicators of Household Social Consumption on Education in India” [17] indicates that only 10.7% of households have computers and 23.8% have internet facilities. Digital literacy in terms of knowledge and ability to operate computers and to use the internet was equally low. The NSS survey also highlighted a significant digital divide in terms of area of residence and socioeconomic categories. In our study, we have found that only 6.2% of households in the study have laptops and 81.5% have availability of internet services. The difference in the data is due to the small sample size and smaller area of data collection.

According to Di Pietro et al. [18], four main conclusions seem to emerge on the possible impact of COVID-19 on education, that is, less time spent in learning, stress, a change in the way student interacts and lack of learning motivation. In our study, we have included the parameters of student interaction and calculated the frequency and percentage. We have included ‘distractions’ in learning instead of ‘learning motivation’. We have collected data regarding the increased stress on the parents instead of the teachers.

The result of the School Education Gateway survey which was conducted from April 2020 to May 2020 attracted 4895 respondents from more than 40 countries, of which 86% were school heads, and 66.9% were teachers who taught online for the first time [19]. Many teachers had problems accessing technology. In our study, we could not include the teachers as no permission was given from the school to share the data regarding the schedule of the online classes and the online attendance of the class. Engle et al. [20] suggest various strategies for promoting equal education in all strata of society.

According to a study by Zahra et al. [21], around 70% of Pakistan's population live in rural areas. In rural areas, there are no facilities. So rural area students face many problems, most of the students drop out of school due to the unavailability of resources and laptop. In our study, we have collected data and conducted a study on online education and its effects on parents and students in an urban slum area.

A study by Fawaz and Samaha [22] focuses on the psychological impacts of the pandemic. Another study by Ma et al. [23] also focuses on this issue. We have not considered the psychological aspects of the online mode of education in our study, however, we have included reduced social interaction and peer interaction of students due to online education in our study.

Our study focuses on the barriers to online education for the school-going children of the urban-slum areas of Pune. Our study focuses on the hindrances in online education like resource scarcity, financial burden over parents, and decreased peer interaction/social interaction. Due to the lower socioeconomic status of the family and the lack of proper resources, the children face problems with online modes of education.

Limitations

The sample size of our study was small as we have chosen only one UHC of the urban slum area of Pune. The time duration for the study was short therefore the evaluation of learning skills, reading, and writing skills of the students were not taken into consideration and were not evaluated.

## Conclusions

The study concludes that students from urban slum areas face problems in attending online classes due to various reasons like lack of high-speed internet services and non-availability of gadgets. The learning of the school-going students affects the learning skills required throughout life. Students and parents from the slums mostly belong to lower socioeconomic classes and lack the motivation to continue learning as a result of their financial crisis. The problems like unavailability of resources need to be addressed. Though there are parents who have smartphones, the lack of motivation and distractions during classes can be a reason for their lower attendance. Online education is a solution to continue education in pandemics and therefore it is necessary to prevent barriers that form inequalities in education.
